# A Digital Innovation for the Personalized Management of Adherence: Analysis of Strengths, Weaknesses, Opportunities, and Threats

**DOI:** 10.3389/fmedt.2020.604183

**Published:** 2020-12-14

**Authors:** Anna-Elisa Hein, Bernard Vrijens, Mickael Hiligsmann

**Affiliations:** ^1^Faculty of Health, Medicine and Life Sciences, Maastricht University, Maastricht, Netherlands; ^2^Faculty of Management, Economics and Social Sciences, University of Cologne, Cologne, Germany; ^3^AARDEX Group, Research and Development, Liège, Belgium; ^4^Department of Public Health, University of Liège, Liège, Belgium; ^5^Department of Health Services Research, Care and Public Health Research Institute (CAPHRI), Maastricht University, Maastricht, Netherlands

**Keywords:** adherence management, medication adherence, patient compliance, precision dosing, digital health, patient-centered care, SWOT-analysis

## Abstract

**Introduction:** Personalized medicine and management of adherence are potential solutions for the suboptimal use of medicines. Digital medication management innovations currently under development combine both aspects. This research aims to investigate facilitators for and barriers to the translation of digital innovations for personalized medicine and adherence management into clinical practice from the policymaker and regulator perspective.

**Methods:** A mixed-method study was used combining a scoping review to identify main interests, semi-structured interviews (*n* = 5) with representatives of European health policymaking and regulatory organizations, and a supplementary literature review to investigate key subthemes. The SWOT analysis was used for the qualitative analysis.

**Results:** The literature reviews and the qualitative interviews suggested that digital solutions can facilitate the personalized management of medications and improve quality and safety, especially as the openness for digital health solutions is increasing. Digital solutions may, on the other hand, add complexity to the treatment, which can be perceived as a potential barrier for their uptake. As more multidisciplinary and participative structures are emerging, digital solutions can promote the implementation of new services. Nevertheless, change progresses slowly in the task-oriented structures of health systems. Integration of digital solutions depends on all stakeholders' willingness and abilities to co-create this change. Patients have different capabilities to self-manage their medical conditions and use digital solutions. Personalization of digital health solutions and integration in existing service structures are crucial to ensure equality among population segments. Developments in the digital infrastructure, although they are partly slow and not well-aligned, enable the implementation of innovations in clinical practice leading to further advances in data generation and usage for future innovations.

**Discussion:** This study suggests that digital solutions have the potential to facilitate high-quality medication management and improve adherence to medications, enable new service structures, and are essential to drive further innovations in health care. Nevertheless, increasing the self-responsibility of patients can have undesirable effects on health outcomes, especially within vulnerable population segments. Digital health solutions can be an opportunity to optimize the use of medicines and thus their efficiency. Well-conceived development and implementation processes are needed to also realize improvements in equality and solidarity within health systems.

## Introduction

Health care systems in Europe and around the globe are facing various challenges such as the increasing incidence of chronic conditions leading to cost constraints. Improving effectiveness, accessibility, and resilience of health care systems with particular attention to medicines are major aspects on the political agenda (1). In this context, voices are raised for a more “responsible use of medicines” aiming to improve clinical benefits while being aware that resources are scarce (2, 3). The importance of administering “the right drug for the right patient at the right time” (4) has been highlighted by initiatives addressing the inadequate and suboptimal use of medication (3, 5, 6).

Combining personalized medicine with adherence management is thus one approach to optimize the use of medicines. The interest in and potential of personalized medicine increase with the advanced understanding of individual factors that influence the onset and progression of diseases (7, 8). Therapeutic drug monitoring and technologies enabling flexible and individualized drug delivery facilitate personalized medication regimen (4). Target concentrations of the drug need to be well-established for each individual patient, and potential comorbidities and polypharmacy have to be taken into consideration. However, this is not always the case, and there is room for improvements to decrease the risk of toxicity and ensure clinical effectiveness for the individual patient (9). A profound understanding of biological, environmental, and behavioral patient characteristics that may lead to variability in drug responses is thus needed to design and execute treatments for the individual patient (10). Medication adherence, defined as “the process by which patients take their medications as prescribed” (11), is an important behavioral patient characteristic. As adherence behaviors currently remain poor and suboptimal, studies have shown that a significant proportion of patients do not take their medications as prescribed and hence experience less beneficial health outcomes (12–15). Furthermore, health systems and societies are confronted with significant inefficiencies, resulting in higher costs and overall financial burden (12–15). As a matter of fact, “if patients do not take their medicines, then there is no action, [and] no benefit” (5). Consequently, non-adherence may lead to potentially harmful overdosages and polypharmacy due to unnecessary escalations in prescribed dosages and further medications added to the regimen (16). Engagement and support of patients to adhere to their medication have been associated with improved clinical benefits and quality of life, as well as the efficiency of health care systems (15, 17, 18). Adherence management should thus be an integral part of personalized medicine to realize its full benefits (10, 11).

Digital solutions in the form of converging technological platforms can have a significant impact on future health care services and therefore fall under the regulation of medical devices (19). Health problems can be approached with multidisciplinary and participative interventions as digital solutions allow for improved information generation and communication leading to more appropriate care and better coordination of services. Digital health solutions in the field of personalized medicine and adherence management can thus facilitate individualized medication regimen and ensure their optimal and effective administration to the patient (20–22).

**Figure 1:**
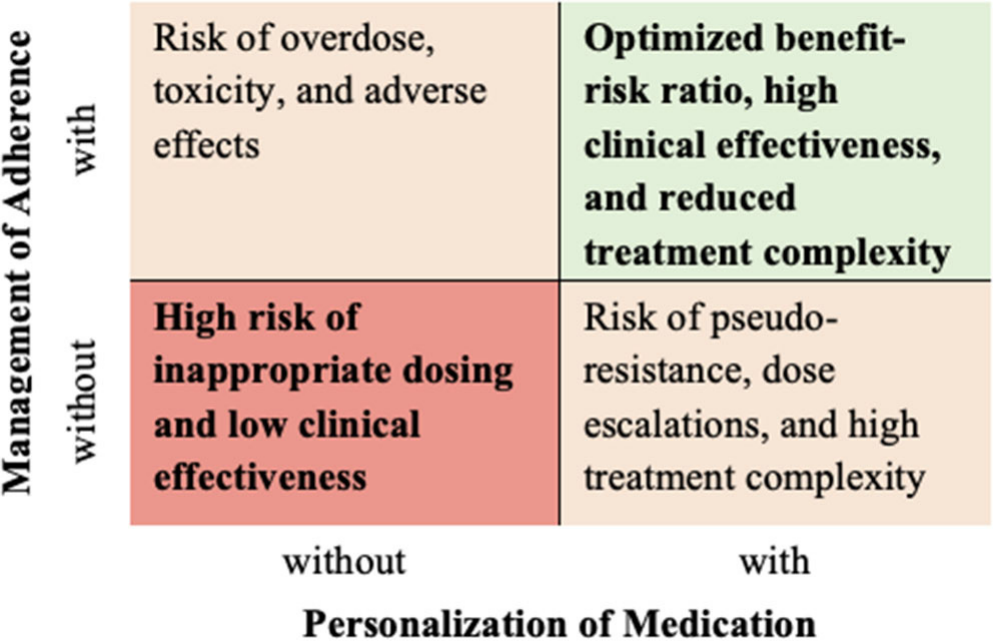
Adherence Management as an Integral Part of Personalized Medicine (own representation).

This research was motivated by a digital health solution that is currently being developed by the AARDEX group, a pioneering company in adherence measurement and management. The digital medication management innovation is based on the assumption that the personalization of medications and the management of adherence are most effective when being addressed simultaneously (Figure 1). Thus, a “precision dosing” feature provides and visualizes real-time information about the patient's position in the therapeutic window. It is based on pharmacometrics models and individual adherence data derived from an electronic medication event monitoring system (MEMS®). Additionally, a person-centered, behavioral adherence management intervention supports the motivation of individuals and improves the habit of medication taking. Personalization to the patient can be achieved based on targeted adherence feedback.

Although the relevance of innovations that combine technological and behavioral strategies to improve the personalization of medications and the management of adherence has been recognized, deployment of these digital solutions in health care systems remains challenging (20). As innovations are changing the way services are provided, they have to satisfy the needs of multiple stakeholders within the health care system such as quality and safety, financial sustainability, and equal access (21, 23). Digital solutions that have a medical purpose such as monitoring and treating diseases thus fall under the category of medical devices, which are highly regulated by market access and reimbursement processes (19, 24). Because of regulators' and policymakers' key role in market authorization and reimbursement processes and implementation, the investigation of facilitators and barriers from the regulator and policymaker perspective is crucial to understand stakeholder expectations and market opportunities and developments. Therefore, the goal of this research is to investigate strengths, weaknesses, opportunities, and threats (SWOT) of digital medication management innovations for the personalization of medications and the patient-centered adherence management from the perspective of European regulators and policymakers.

## Materials and Methods

### Study Design

The research is of qualitative nature and is based on the grounded theory approach as the established framework is the result of an inductive reasoning process (25). Two literature reviews and semi-structured interviews were combined. A scoping review was first conducted to conceptualize major fields of interests of regulators and policymakers. Then, semi-structured interviews were performed to investigate and analyze SWOT within each field of interest. The SWOT analysis is a tool for the identification of environmental relationships and complex strategic decision making and has been applied in the health care sector to identify internal and external subthemes and their interdependencies (26, 27). The SWOT analysis was chosen as an underlying framework to organize internal strengths and weaknesses and external opportunities and threats for the digital medication management innovation for the personalization of medications and the patient-centered adherence management and similar digital solutions from the perspective of regulators and policymakers. Key subthemes that were identified within the interviews were further investigated within a supplementary literature review. Ethical approval was obtained from the Maastricht University ethical board at the Faculty of Health, Medicine and Life Science (REC no. FHML/HPIM/2020.079).

### Scoping Review

#### Data Collection

A scoping literature review was conducted to conceptualize major fields of interests of policymakers and regulators in the area of medical devices around personalized medicine and adherence management. The market access and reimbursement processes for medical devices and strategic approaches for medicines research and development served as a basis. PubMed was searched for “medical device” or “medical device regulation” combined with “Europe,” “authorization,” “reimbursement,” and related terms. Only documents in English were included. Because of recent changes in regulations from the Medical Devices Directive to the Medical Device Regulation (MDR) (19), the search was limited to articles published within the last 5 years (Supplementary Material A). Furthermore, strategic reports, position papers, and documents concerned with personalized medicine, adherence and empowerment, and digital health in Europe were searched to identify further interests and strategic directions not captured by the European-wide MDR and national procedures. Reports and documents of institutions such as the European Commission, the European Alliance for Personalized Medicine, the International Consortium for Personalized Medicine, Digital Health Europe, the European Federation for Pharmaceutical Science, and the European Federation of Pharmaceutical Industries and Associations were thus searched and identified.

#### Data Analysis

Literature investigating the actual interests and preferences of regulators and policymakers regarding development, market approval, reimbursement, and patient access procedures of medical devices were included for the data analysis. Documents identified in the PubMed search were not included if they discussed potential future regulatory and policy directions or regulations and policies outside the European Union or investigated the effect of regulations and policies on specific medical devices. Strategic documents and position papers were included if the strategic direction was still in force and focused on medicines and medical devices research, the digital transformation of health care, or the personalization of treatments. Data were extracted according to the different themes that are relevant during the processes medical devices have to go through before they are translated into practice at the medical encounter. Two major fields of interest thus emerged around the market access processes at the European level as well as the reimbursement and pricing processes at national levels. A third field of interest emerged around economic growth and competitiveness. These themes were used as the extraction structure for the semi-structured interviews in the following.

### Semi-structured Interviews

#### Participants

Semi-structured video interviews were performed in May 2020 with representatives of organizations in the regulatory and policymaking field and researchers informing these policymakers. The convenient sampling approach was used, which led to a sample of five experts and was limited to four western European counties. Interviewees were recruited from the wider network of BV who also made the first contact. AH, who conducted the interviews, did not have any relationship with the participants before the study started. Interviewees were informed that the interviews are part of her master thesis placement at the AARDEX Group. Experts with different roles and responsibilities in the regulatory and policymaking processes related to medication or medical devices on the European or national level were found eligible. Seven participants were invited to participate in the research via email. Five experts from four countries replied and agreed to the interviews. All participants provided written informed consent. The selection of interview partners ensured that various perspectives along the regulatory and policymaking processes in multiple European countries are reflected.

#### Data Collection

Each participant was interviewed by AH according to an interview guide. This study was conducted within her thesis project in the M.Sc. Program Health Care Policy, Innovation, and Management at Maastricht University. AH had basic experience in conducting semi-structured interviews. The interview structure and questions were developed according to Tolley et al. (28) field guide for qualitative research. The interview guide was pilot tested between AH and BV and was adjusted for each interviewee depending on their background and expertise. The interviews consisted of two main parts (Supplementary Material B). Within the first part, interviewees were asked to emphasize their priorities in regulatory and policymaking processes to understand their backgrounds and perspectives. The second part started with a short introduction of the digital medication management innovation for the personalization of medications and patient-centered adherence management. The analysis of SWOT (26) was used as an underlying structure for the ensuing questions relating to the introduced innovation and similar digital solutions. As the health care industry is characterized by a dynamic environment and interactions of the quadrants (27), the interview was structured according to the two main categories of the SWOT analysis: first, the internal category with (a) strengths (helpful) and (b) weaknesses (not helpful) and, second, the external category with (c) opportunities (helpful) and (d) threats (not helpful).

#### Data Analysis

Interviews lasted on average about 50 min and were recorded and transcribed by AH, who also took field notes during the interviews. The language used was English except in one case (German). Subthemes were identified as they were mentioned repeatedly by multiple interviewees. The level of data saturation was discussed to be sufficient to establish a framework between AH and BV as no new major subthemes emerged within the interviews. Participants had the opportunity to check the transcripts for their correctness and comment to clarify ambiguities. The validated transcripts served as the basis for the data analysis.

AH analyzed and coded the interview transcripts using the NVivo Software (QSR International Pty Ltd., version 12). A first analysis was conducted by identifying as many codes as relevant and assigning them as either helpful or not helpful. Thus, strengths and opportunities were distinguished from weaknesses and threats from the regulator or policymaker perspective. The themes that were identified within the initial literature search served as the basis for the second step. All codes were allocated to the following fields of interest: (a) quality, safety, and effective patient access to personalized medicine; (b) new services structures, person-centeredness, and allocation of resources; and (c) innovation, competitiveness, economic growth, and digital infrastructure. Codes were then distinguished by internal concepts that directly relate to the integration of digital solutions for personalized medications and adherence management and concepts that influence the uptake and implementation of these digital solutions through external developments and expectations. Hence, subthemes were identified within the fields of interest as either strengths, weaknesses, opportunities, or threats. Quotations are presented anonymously to illustrate the findings. Appropriate reporting of the study design, analysis, and findings was ensured using the COREQ checklist (29). The results were checked and discussed between AH and BV. Interviewees were given the opportunity to comment on the results.

### Supplementary Literature Review

#### Data Collection

A supplementary literature review was finally performed to get a more detailed understanding of key subthemes identified within the SWOT analysis. Regulators and policymakers expressed a high interest in the topics of patients' abilities and willingness to be self-responsible for their health and self-manage medications. While equal and effective patient access to high-quality health services emerged as an important interest, experts seem to have different priorities relating to consequences on equality and accessibility by increasing the level of self-responsibility of patients and the role of digital self-management solutions in this matter. A PubMed search was thus performed to understand, on the one hand, barriers and facilitators and differences among subpopulations relating to the usage of digital self-management solutions and, on the other hand, approaches to prevent unequal accessibility and utilization when implementing such solutions in clinical practice. The terms (and synonyms and related terms) “self-management,” “usage,” “accessibility,” and “eHealth” were combined in the search (Supplementary Material A). Only documents in English were included. Because of the recency of digital self-management solutions, the search was limited to articles published within the last 5 years.

#### Data Analysis

Studies that identified barriers and facilitators associated with different levels of acceptability, utilization, or willingness to use digital health solutions for the self-management, empowerment, and education of patients were selected. Furthermore, studies were included in the analysis if they discovered approaches to address these differences. Findings were analyzed in the context of the previously developed SWOT analysis. Data were extracted according to the widely used rainbow model of the main determinants of health by Dahlgren and Whitehead (30). The model defines “general socioeconomic, cultural, and environmental conditions,” “living and working conditions,” “social and community networks,” “individual lifestyle factors,” and “age, sex, and constitutional factors” as layers of determinants. The framework was used to provide an overview of causes for inequalities in the realization of health benefits by digital health solutions identified in the included studies. Moreover, data relating to development, design, and implementation requirements to address inequalities were extracted.

## Results

### Scoping Review

The initial scoping literature search led to 38 results in PubMed, which were assessed based on title and abstract. Seven articles were included in the analysis (24, 31–36). Within the additional search for strategic articles, six documents were identified based on the proposed strategies' intent and time frame (6, 21, 22, 37–39). The analysis resulted in an overview of interests and strategic objectives of policymakers and regulators relating to medical devices and medicines. Three main fields of interest were identified and used for data reporting: (a) quality, safety, and effective patient access to personalized medicine; (b) new services structures, person-centeredness, and allocation of resources; and (c) innovation, competitiveness, economic growth, and digital infrastructure.

### Quality, Safety, and Effective Patient Access to Personalized Medicine

Regulators and policymakers aim to ensure effective patient access to high-quality and safe health services (24). Therefore, the market approval of medical devices is regulated by the MDR of the European Commission. Medical devices are granted market access in the form of the Conformité Européenne (CE mark) if clinical safety and performance have been proven (19, 35). A general orientation toward quality improvements and treatment personalization can be observed with the aim of achieving better clinical outcomes (32). To accommodate individual patient needs, reduce the risk of adverse effects, and improve the efficacy, a system approach, including personalized medicine and complex disease-modifying drug treatments, is proposed (22). Digital solutions can support these new care models, and it is thus of interest to deploy them throughout health systems and ensure their accessibility and uptake of all population segments (21). Regulators and policymakers strive to generate high-quality health data and its use in personalized, evidence-based treatments to optimize patient care on a scientific basis (21, 37, 39).

### New Services Structures, Person-Centeredness, and Allocation of Resources

To ensure the sustainability of health systems throughout Europe, efficient allocation decisions have to be made while promoting innovations that lead to improved and holistic service delivery (32). While reimbursement and pricing decisions are made on the national level (24), broader strategic directions and approaches are formulated at the European level (21). For medical devices, countries apply different procedures and criteria and involve various agencies in the decision-making process (24, 31, 33, 36). As national regulators and policymakers are concerned with allocation decisions, cost-effective use of resources and cost control can be seen as major interests besides clinical effectiveness and accessibility (24, 31, 32). Frequently applied assessment elements are safety, effectiveness, and efficiency, costs, and economic consequences. Furthermore, social, organizational, ethical, and legal aspects are often considered (33, 34, 36). Strategic directions for regulations and policies on the European and national levels reflect the aim to design purposeful and sustainable health systems and avoid financial waste (21, 22). Highly recurrent approaches that are assumed to improve the financial effectiveness are a paradigm shift toward health promotion, disease prevention, and patient-centeredness. Different strategies are proposed to reach these goals. Patient empowerment and self-responsibility, well-equipped health care professionals, multidisciplinary and integrated care models, and the implementation of new technological and digital solutions are assumed to be key enabler for holistic, cost-effective, and person-centered care provision and are thus important interests of regulators and policymakers (6, 21, 22, 37–39).

### Innovation, Competitiveness, Economic Growth, and Digital Infrastructure

Besides the accommodation of patients' and society's health-related needs, innovations in health care have a sizable economic dimension. Generating economic value and remaining competitive are thus further major interests (6, 22). Enabling technologies are an essential lever and key for maximizing the research and discovery potential and the development of new health care models and personalized medicine. Active promotion of research and innovation can stimulate economic growth and establish an industrial leadership of Europe. International co-operation and collaboration within Europe in data generation, management, and governance, interoperability of systems, and implementation of novel technologies can generate a significant competitive advantage in the digital transformation of health systems and the realization of clinical and financial benefits associated with new care models (6, 21, 22, 37–39).

### SWOT Analysis

Five interviews were conducted with experts (two females, three males) from four European countries (Belgium, Germany, Luxemburg, the Netherlands). As no new major subthemes emerged within the interviews, a level of data saturation was reached that allowed to establish the framework. The experts are active in different regulatory agencies, governmental bodies, research departments, and organizations concerned with the design of health systems. One interviewee is working in a health ministry and is concerned with pharmaceutical care focusing on hospital pharmacies and seamless care after discharge. Another interviewee is working in a governmental body directly under the control of a health ministry in the field of medicines and pharmacy. A third interviewee works in the clinical assessment of medicines and medical devices and gives advice on the national and international level. This interviewee also holds a position as a researcher in a related field. The fourth interviewee is a researcher and concerned with the impact of policy decisions on health and the safe use of medicines. The last interviewee is an expert in health systems research, health policy design, innovations implementation, and health economics. All interviewees have experiences with the design, implementation, use, or evaluation of digital solutions in medicines or medical devices. Clinical effectiveness, patient safety, and quality of care are a major field of attention for four interviewees. Health economic aspects and financial sustainability of health systems and policies are important themes for two interviewees.

The analysis of the priorities of regulators and policymakers confirmed the three major fields of interest identified in the scoping literature review to a large part. Depending on the responsibilities of the interviewees, different interests were emphasized. Improving the benefit–risk ratio of medications, ensuring patient access to medications, cost-effectiveness, multidisciplinary approaches, and data governance emerged as the most important interests. Promoting personalized medicine and proactive service structures were mentioned as important aspects to improve health services but were not stated as top priorities. Strategic aims such as competitiveness and economic growth were not explicitly mentioned as goals. The SWOT analysis resulted in 7 strengths, 8 weaknesses, 11 opportunities, and 10 threats, as shown in Tables 1, 2 (see Supplementary Material C for more details). In the following, the results are described per major field of interest, starting with the external threats and opportunities and followed by internal strengths and weaknesses.

**Table 1:** Strengths and weaknesses of digital innovations for the personalization of medications and the management of adherence.

**Strengths**	**Weaknesses**
*Quality, safety, and effective patient access to personalized medicine*	
•Facilitates better management of illnesses and medication and can reduce treatment complexity through personalization and information •Improves quality, safety, effectiveness, and benefit–risk ratio of medications through personalization and feedback	•May add complexity to treatments and the management of medication •May only be perceived necessary for a limited number of medications •Potential exclusion of (vulnerable) patient populations from the optimal use of medicines
*New services structures, person-centeredness, and allocation of resources*	
•Promotes patient-centered and multidisciplinary service provision and a new way of working between health professionals •Facilitates a participative way of service provision and motivates patients to take an active role •Increases self-responsibility and capabilities of patients to understand and self-manage their medication through empowerment, education, and support •Improves cost-effectiveness by reducing economic burden related to ineffective management of medication	•Ability to be self-responsible for medication management differs among patients •Unclear preferences regarding the value of new technologies and hard to ensure their appropriate use •May disturb the relationship between health care professionals and patients •Implementation costs may exceed cost savings, and integration requires additional training and collaborative efforts
*Innovation, competitiveness, economic growth, and digital infrastructure*	
•Generates valuable data for improvements and further innovations	•Generated data may have irregularities that are hard to explain

**Table 2:** Opportunities and threats of digital innovations for the personalization of medications and the management of adherence.

**Opportunities**	**Threats**
*Quality, safety, and effective patient access to personalized medicine*	
•Increasing openness toward approaches to personalize treatments and development of supportive digital solutions •Increasing demand to improve adherence to medications with a narrow therapeutic window	•High complexity of medications and uniqueness of patients •Suboptimal adherence behavior in treatments with a narrow therapeutic window leading to considerable disease-related consequences and adverse effects
*New services structures, person-centeredness, and allocation of resources*	
•New forms of collaboration and multidisciplinary approaches are emerging •Service structures to ensure long-term sustainability and patient-centeredness are gaining importance •Focus on methods to allocate financial resources in a cost-effective manner •Increasing openness for digital solutions in health care •Many patients are willing and able to self-manage their therapies and demand empowerment and participation •Affinity of many patients for digital technologies, wearables, tracking of individual data	•Task-oriented service and remuneration structure and no clear strategy to improve and implement multidisciplinary and patient-centered structures •Passive role of the patient with insufficient health literacy, self-responsibility, and ability to self-manage medications •Determinants of adherence are complex and awareness among health care professionals of non-adherence is low •Conservative attitude toward digitalization among many health professionals and slow implementation processes •Scarcity of financial resources, increasing complexity and costs of care, and low willingness to pay for new care models
*Innovation, competitiveness, economic growth, and digital infrastructure*	
•Ongoing improvements of the digital infrastructure and implementation processes for digital health solutions •Initiatives to generate health data and make best use of available sources •Opportunity of certification and development of registries to ensure high-quality applications, to increase visibility, and to guide uptake	•Slow progress in the infrastructure for digital solutions and data governance regulations •Highly regulated and bureaucratic market access •Insufficient generation and usage of health (and adherence) data

### Quality, Safety, and Effective Patient Access to Personalized Medicine

The rising complexity of medications and the development of high-risk drugs can threaten the quality and safety associated with treatments. Especially experts with a background in pharmacy and a strong focus on regulatory decisions and their impact on patients' health highlighted the following subthemes. Complex medications with narrow therapeutic windows are a challenge for health care professionals as the uniqueness of patients and their pathology demand them to achieve individual concentrations for each patient that are safe and effective. Furthermore, patients' suboptimal adherence behavior to these medications threatens attempts to improve quality, safety, and personalization of treatments as it may lead to considerable disease-related consequences and adverse effects. Opportunities to meet these challenges are seen in the personalization of medicine, and the use of supportive digital solutions that help health care professionals ensures the right administration of the drug, promotes effective communication along the care chain, and enables patients to manage their conditions. The increasing awareness of the negative consequences of non-adherence is further supporting initiatives aiming to improve adherence, especially for high-risk medications. Digital solutions for medication individualization and the personalized management of medication adherence can add value as they can facilitate better management of illnesses and medication. Personalized information about the drug, such as target concentrations or adverse effects, can help patients manage the medication and avoid adverse effects. Further, the complexity of treatments may be reduced by such digital solutions through the alignment of multiple medications. Participants also mentioned the potential to improve quality, safety, effectiveness, and the benefit–risk ratio of medications as a significant strength of personalizing medicines and promoting adherence:

“*You get the right amount of drug for the right type of patient, making sure that you get the best clinical benefits out of the drug and [are] still within a range in the clinical therapeutic window, that you don't see harms. I think that is the main advantage that [you] could expect of this. And with this stimulation of adherence, obviously, you also stimulate that these benefits are really achieved.”*

Nevertheless, the complexity of treatments and medication management could also increase through the additional application. Improvements in medication research and development currently lead to less severe or perceptible adverse effects or reduced treatment complexity. Hence, although digital solutions for the personalization of medications and patient-centered adherence management may improve quality and safety of many medications, the necessity is perceived for only a limited number of drugs with narrow therapeutic windows. One interviewee stated:

“*You have to think that through every time—do I need all this kind of extra information to use my medicine?”*

Furthermore, the use of the digital solution itself and the information provided may demand too much from some patients and exclude especially vulnerable population segments from the optimal use of medicines.

### New Services Structures, Person-Centeredness, and Allocation of Resources

The current task-oriented structure of health care systems, with an organization of health services and remuneration schemes that disincentivize a proactive and multidisciplinary care provision, is a significant threat to the effective allocation of resources and the implementation of new, person-centered service structures. All interviewees mentioned forms of inertia to change the structure of health service provision as a major barrier to the implementation of person-centered, holistic, and more preventive approaches. Because of these classical service structures, many patients are not assuming self-responsibility for their health, having a rather passive role in the treatment process, and a low ability to self-manage medications. Moreover, low levels of health literacy among patients lead to insufficient awareness of behavior related to health and therapies and thus low demand for education and empowerment. Furthermore, the task-oriented service provision limits health care professionals to detect and address the underlying causes of non-adherence. More holistic routines are needed to raise providers' awareness of the importance of improving adherence. The conservative attitude toward digital solutions among many health professionals was mentioned as another barrier that slows down the transformation of health services.

“*Many health care professionals are rather conservative in the way of approaching [digital] care.”*

Especially the older generation of health care professionals is not trained for the use of digital solutions. Different mindsets regarding the digital transformation of health care coexist among professionals, and in combination with the availability of many low-quality applications, this may lead to mistrust in digital health solutions.

“*But there is also a fear. So, I think physicians also need to be reassured that the app is built on reliable data because we all know this garbage in garbage out from all these applications.”*

Especially the uptake of solutions aiming to improve multidisciplinary approaches that rely on reliable communication of all professionals in the care chain may be complicated by these aspects. Rising health care costs and scarcity of financial resources further lead to a low willingness to pay for additional technologies or services. A proactive and preventive orientation demands payers to invest in new person-centered and multidisciplinary structures without having guaranteed costs-savings.

“*There is less money which is spent on prevention than on a curative approach.”*

The scarcity of resources may thus also lead to less solidarity and more self-responsibility of citizens to invest in their health and prevent or manage diseases. Despite these barriers to restructuring health care, especially experts in the field of policymaking mentioned the need to establish more person-centered and multidisciplinary care chains to satisfy patient needs and build more sustainable health systems. New forms of collaboration are emerging as opportunities to make the best use of available resources and provide holistic care. As stated by one interviewee:

“*There is nothing cheaper than good care, and there is nothing more expensive than poor care.”*

The importance of new patient-centered service structures is thus increasing. Proactive measures and preventive approaches are more and more promoted, as they are likely to be more cost-effective in the long term. Cost-effectiveness is an important criterion to ensure financial sustainability. Thus, Health Technology Assessment (HTA) and regulatory agencies collaborate in the alignment of HTA processes and regulations of new service models to improve implementation and reimbursement procedures. Furthermore, the COVID-19 (coronavirus disease 2019) pandemic has shown how fast digital solutions can be implemented in health care. The uptake of digital health solutions has accelerated, and health care providers and patients are making positive experiences. Besides these recent developments, directions toward self-management, empowerment, and a participative way of service provision are promoted, and many patients are willing and able to be more self-responsible and take an active role in their treatments. Additionally, the majority of citizens have an affinity for digital technologies such as wearables or apps to track individual health measures:

“*I think the people like more and more to measures things themselves. To know about their health, people are having Apple watches, you know. It's more and more common, and I think it'll be more and more common to have these health apps.”*

It can thus be assumed that these citizens have a positive attitude toward digital solutions to support their treatments.

Significant strengths of digital solutions for the personalization of medications and the patient-centered adherence management relate to these opportunities. Interviewees stated that they can promote new ways of working between health professionals and person-centeredness by coordinating collaborative efforts of multiple professions and connecting relevant patient information. Furthermore, they facilitate a more participative approach and give patients a more active role in the treatment. Improved access to and sharing of health data, as well as appropriate communication and feedback, can enable patients and providers to make decisions jointly. Digital solutions not only can facilitate more involvement at the medical encounter but also can increase the health-related self-responsibility of citizens and the capabilities of patients to understand and self-manage their medication. Empowerment through personalized education, visualization of adherence and health data, and support in building habits and routines can increase the ability to make decisions and manage challenging health conditions. Experts emphasized the importance of user-friendliness and accessibility to realize these benefits for as many persons as possible. If digital solutions successfully facilitate new care models in the form of multidisciplinary and person-centered approaches, interviewees expect them to improve the cost-effectiveness of medications through fewer costs associated with their suboptimal use and non-adherence. However, there are also weaknesses associated with digital solutions as the ability and willingness of patients to be self-responsible differ and highly depend on various patient characteristics. Not all patients can understand the interrelationships of their health, medication, and behavioral aspects or are unfamiliar with digital devices and apps. Especially vulnerable patient groups may not benefit from this form of digital support. Among those patients who are able and willing to use digital health solutions, interviewees added for consideration that preferences, the perceived user-friendliness, and also the adherence to the digital solutions may vary. These differences make it hard to ensure their appropriate use and thus hamper the realization of benefits. Furthermore, the traditionally confidential relationship between health care professionals and patients could be disturbed. The implementation of holistic digital solutions is also associated with additional costs. Besides the costs of the applications, integration requires additional efforts relating to new reimbursement schemes, training of professionals, and collaborative efforts between them. Hence, integration demands scarce financial and human resources.

### Innovation, Competitiveness, Economic Growth, and Digital Infrastructure

The implementation and utilization of digital solutions in health care rely on a profound information technology (IT) infrastructure and clear data governance. Slow progress and fragmentation are seen as an essential threat by the interviewees. Especially experts who are active in health policymaking saw further barriers in the bureaucratic market access of new technologies.

“*I think societies or health care systems are ready for innovations, but I think the bureaucracy and the regulations slow them down. Due to all the steps that you need to take to get the innovations really implemented, for some they are already outdated when you are at the end of that row.”*

Most interviewees mentioned insufficient data generation tools and suboptimal usage of available health data as a considerable challenge to raise awareness and prove the importance of implementing new care models supported by digital solutions. Nevertheless, opportunities lie in current developments and improvements in the IT infrastructure. Digital solutions are being implemented at a small scale or in pilot projects, and integration processes are improving. Further, many initiatives tackle the challenges of suboptimal data generation and usage. Patient registries, real-world evidence, the input of wearables and apps, or information patients have to provide if they receive costly treatments are some sources to generate high-quality data and increase the understanding and awareness of non-adherence and other reasons behind the suboptimal use of medications. Besides that, interviewees working in health policymaking saw a notable opportunity in certifications for digital solutions and registries to guide the uptake of such novelties. The CE mark or national registries can reassure health care professionals that the app complies with all regulations and provides high-quality and safe services.

A major strength of digital solutions for personalizing medicines and managing adherence is thus the generation of high-quality individual health data. These data can be used to raise awareness of problems such as non-adherence and promote further improvements in therapies and the development of innovations. On the downside, data generated by applications at the patients' home may have irregularities that are hard to explain:

“*How do you ensure that the patterns that you see are true? Because people also do the strangest things, you know. They open the bottle and take seven doses out and then leave it closed for a week and then it looks like they are not doing what they are supposed to, but they really are.”*

Thus, the validity of the data and information generated by patients through digital solutions must be approached with caution.

Overall, participants were optimistic that digital health solutions will be one facilitator of new personalized and multidisciplinary service structures in health systems that can ensure sustainable allocation of resources and satisfy the needs of all stakeholders more optimally than the current system.

“*[New approaches] will evolve for more patient-focusing aspects with an economic care model that can be available and affordable for everybody.”*

This statement highlights the importance of ensuring that all population segments benefit from new ways of service provision. While initiatives to increase patient empowerment, education, and self-responsibility were generally valued and in line with the focus on preventive and proactive health services, concerns around equality, fairness, and solidarity were raised. Further, the strength of empowering patients and giving them a more active role in their treatment was especially discussed against the weakness of potentially excluding vulnerable groups from the optimal use of therapies. These subthemes were identified as a key topic because the digital medication management innovation for the personalization of medications and person-centered adherence management relies on patients' ability and willingness to become more self-responsible. Although the intervention aims to empower patients in a personalized way to facilitate behavioral change and self-manage their medications, the results of the SWOT analysis suggest that inequalities may arise. A profound understanding of reasons for inequalities and approaches ensuring equality and accessibility among all population segments is thus essential.

Supplementary Literature Review Focused on Digital Self-Management Solutions and Inequalities.

The supplementary literature review was thus conducted to first explore barriers to and facilitators for the acceptance and utilization of digital health solutions focusing on self-management, empowerment, and education. Second, features and requirements to prevent unequal access and usage of these technologies were investigated. The PubMed search led to 35 results, of which 16 articles were included for analysis after the title and abstract assessment (Supplementary Material A). Nine of the identified studies focused on self-management support (40–48), two were related to health education and information (49, 50), and five studies investigated digital health services (51, 52) or health promotion and disease prevention interventions (53–55).

Barriers to and facilitators of acceptance and utilization were explored in 10 studies (40, 44, 47, 48, 50–55), and findings added mainly to the opportunities and threats of the SWOT analysis. All 16 studies investigated approaches to improve equal acceptability and utilization throughout all population segments. These approaches overlap with suggestions made by some interviewees. Successful realization of these approaches adds to the strengths, whereas failure to incorporate them in digital solutions adds to weaknesses. Key findings are summarized in Table 3.

**Table 3:** Barriers and facilitators for acceptance and utilization of digital health solutions and features and requirements to promote equal access and usage among all population segments.

**Layer (30)**	**Barriers and facilitators for acceptance and utilization of digital health solutions**	**Features and requirements to promote equality in access and usage**
General socioeconomic, cultural, and environmental conditions	•Race and ethnicity •Income levels •Socioeconomic status	•Tailor the digital solution to cultural aspects and offer the interventions in multiple languages
Living and working conditions	•Education •Retirement and unemployment •Competing life priorities •Access to health services and health insurance status •Internet access, availability of digital devices, and digital literacy •Familiarity or previous experience with digital health	•Present information according to the target population's level of education and health literacy •Complexity and amount of information provided should be customizable •Increase accessibility and ownership of devices and make optimal usage of technology that is already available and used •Increase awareness and familiarity
Social and community networks	•Marital status	•Recognize social network as valuable resources to bridge digital skills or to provide support
Individual lifestyle factors	•General health status	•Tailor the interventions around individual health needs and diseases
Age, sex, and constitutional factors	•Age	•Tailor the interventions to individual needs and capabilities

Results suggest that various determinants lead to different levels of acceptability and utilization of digital solutions for patient self-management, empowerment, and education. Within the layer of “general socioeconomic, cultural, and environmental conditions,” race and ethnicity emerged as one determinant. Members of minorities were found to engage less with digital health solutions. Nevertheless, these results were moderated by internet access (53) and could be explained by lower smartphone ownership (48), or race and ethnicity were used as a potential explanation for the comparable low response rate in a study with a health promotion focus (55). One review reported that efforts made to design digital health solutions in a culturally appropriate way may explain the findings that no differences appeared between different ethnic groups (40). Lower income levels were identified as another possible determinant for less engagement in digital solutions or reduced willingness to use them (44, 51), and one study reported significant results (54). One study could explain the lower utilization levels with lower smartphone ownership among those with low income levels (48). In another review, one article reported affordability problems related to the devices needed for the digital health solution, whereas other included studies found lower income to be a facilitator for engagement in digital solutions (47). Low socioeconomic status was found to be attributed to less utilization of digital health solutions in one review (40), whereas age appeared as a moderator for this association in another analysis (47). Within the layer of “living and working conditions,” education emerged as the most important determinant. Eight studies found that lower educational levels were associated with less utilization or willingness to engage in digital health solutions. The result in one study were moderated by adjusting for internet access (53) and could be explained by less internet usage in another study (50). One article identified low educational level as a barrier to participate in their study (55). The majority of articles reported a strong and significant association between high educational levels and increased uptake of digital solutions (44, 47, 48, 51, 54). Retirement and unemployment were identified as other barriers (51, 55), whereas internet access served as a moderator for this finding in one study (53). Living with children was found to be facilitators for the uptake of digital solution that shifted a part of the care chain to online services (51), but lack of child care, time, and private space and demanding work schedules were found to be barriers to digital health solutions for self-management or health promotion (40, 55). Further, population segments with limited access to health services (40) or low health insurance status (48) showed lower acceptability of digital solutions than those with good health service accessibility and health insurance status. Limited internet access or availability of digital devices (47, 48, 50, 53) and lower IT skills (51) going along with low digital literacy were found to be related to lower engagement in digital health solutions. Familiarity or previous experience with digital health was found to facilitate the utilization of other digital health solutions (51, 52). However, reluctance to engage in digital solutions was found to be higher when provider did not recommend it (47). Within the “social and community networks” layer, marriage was discussed as a determinant, results were moderated by adjusting for internet access (53). The general health status emerged as a major determinant within the layer of “individual lifestyle factors.” Deteriorations in health, often associated with hospitalization and routine changes, being in a treatment phase and high levels of fatigue and psychosis were associated with low levels of engagement (47, 54). Another study could explain lower utilization with low internet usage among individuals with worse generally health status (50). However, a high perceived risk of illness (40), greater disability and mental problems (47), and being in a post-treatment phase (54) were linked with higher utilization of digital solutions. The presence of chronic conditions may also lead to increased engagement (51). Within the layer of “age, sex, and constitutional factors,” age emerged as an important determinant for the level of engagement in digital health solutions. Older individuals may show lower utilization rates (44, 51). The association between older age and less engagement in digital solutions could be explained by lower rates of internet usage and access (50, 53) or smartphone ownership (48) that one study found a significant relationship (54).

Studies report various approaches to address these different determinants. It was frequently suggested to involve users in the development process (40, 41, 44, 46, 48, 52) and conduct research on characteristics and preferences of the target population, as well as building the intervention on solid theories (40, 41, 43, 46, 50, 54). The potential to address inequalities in health with digital solutions for patient self-management, empowerment, and education highly depends on the acceptability of the technology among the target population (43, 49, 55). Approaches to increase the acceptability and promote utilization among all population segments suggest addressing the previously reported determinants. Within the layer of “general socioeconomic, cultural, and environmental conditions,” tailoring the digital solution to cultural aspects and offering the interventions in multiple languages emerged as a promising approach (40, 43, 46, 49). Results suggest that the information has to be presented according to the target population's level of education and health literacy. The complexity and amount of information provided should be customizable, so individuals neither are overwhelmed nor perceive the content as irrelevant (40, 41, 43, 44, 46, 49, 51). Further strategies within this layer address the limited internet access or availability of digital devices, as well as lower IT skills and digital literacy. While some studies suggest promoting efforts to increase accessibility and ownership (46, 50, 51, 53), others recommend making optimal usage of technology that is already available and used (42, 43, 45, 50, 54). As familiarity and previous experience with digital health solutions were identified as one main facilitator for the utilization of further digital solutions, approaches to increase awareness and familiarity are suggested. Recommended strategies include cost-free opportunities for patients, courses introducing the functions of digital health solutions, and providing support, as well as integrating health care personnel in the intervention to overcome mistrust and increase the reputation (42, 43, 50–52, 55). Within the layer of “social and community networks,” family members, informal caregivers, and the community were identified as valuable resources either to bridge digital skills (42, 46) or to support patients by monitoring their progress and motivating them (45). The general health status emerged as an essential determinant in the layer of “individual lifestyle factors.” Results suggest that patients and health professionals should be enabled to tailor the interventions around individual health needs and preferences, which requires a profound understanding of the needs and routines of the target population and room for personalizing the interventions itself, as well as features such as reminders or motivators (40–47, 55). In some health conditions, empowering, and monitoring features may lead to increased disease distress or be perceived as a control measure. Thus, special attention is required in the design of digital solutions for psychologically vulnerable patient populations (42, 47). Furthermore, interventions should be agile so they can be adjusted to deteriorations in health, periods of hospitalization, and new treatment goals (45, 47). Age is found to be a determinant for the uptake of digital solutions within the layer of “age, sex, and constitutional factors” and is subject to approaches that suggest tailoring the interventions to individual needs and capabilities (40, 48, 49, 51). Besides age, other individual characteristics are important for a good user-experience and user-friendly design. A simple and intuitive interface and personalized feedback, reminders, and trackers were mentioned besides the demand for customization options of the font size and colors as well as the option for a more gamified design including avatars and reward systems (41, 43, 45–47, 49).

## Discussion

Two literature reviews and expert interviews were conducted to investigate SWOT of the digital medication management innovation for the personalization of medications and the patient-centered adherence management and digital solutions of a similar type from the perspective of European regulators and policymakers. Main fields of interests of regulators and policymakers emerged around quality, safety, and effective patient access to personalized medicine, as well as new services structures, person-centeredness, and allocation of resources and innovation, competitiveness, economic growth, and digital infrastructure. SWOT were classified within these fields of interest. Although historically grown structures and associated remunerations schemes, conservative attitudes of professionals and patients, insufficient digital infrastructure, and bureaucratic regulations may slow down change processes, interviewees were optimistic that progressive structures will evolve in the long term. Patient empowerment, education, and self-responsibility and directions toward more preventive and proactive health services emerged as key future directions, but concerns around equality, fairness, and solidarity were raised, and the potential exclusion of vulnerable groups from the optimal use of therapies was discussed. The supplementary literature review added to this key topic and can thus be linked to findings of the interviews relating to the ability and willingness of patients to be self-responsible for their health and self-manage their medications.

To discuss these interdependencies, the adapted SWOT analysis of van Wijngaarden et al. (27) used, which recognizes the complex context and dynamics of the health care environment. In their revised version of the analysis, interactions of the four quadrants are recognized, and classification as a strength or weakness and opportunity or threat is less strict. Instead, three dimensions are identified: the expectations of stakeholders and contextual factors as external elements and resources as an internal element. A profound understanding of stakeholder expectations, contextual factors, and internal resources can thus provide valuable insights into current and future facilitators for and barriers to the digital medication management innovation for the personalization of medications and the patient-centered adherence management and similar digital solutions.

**Figure 2:**
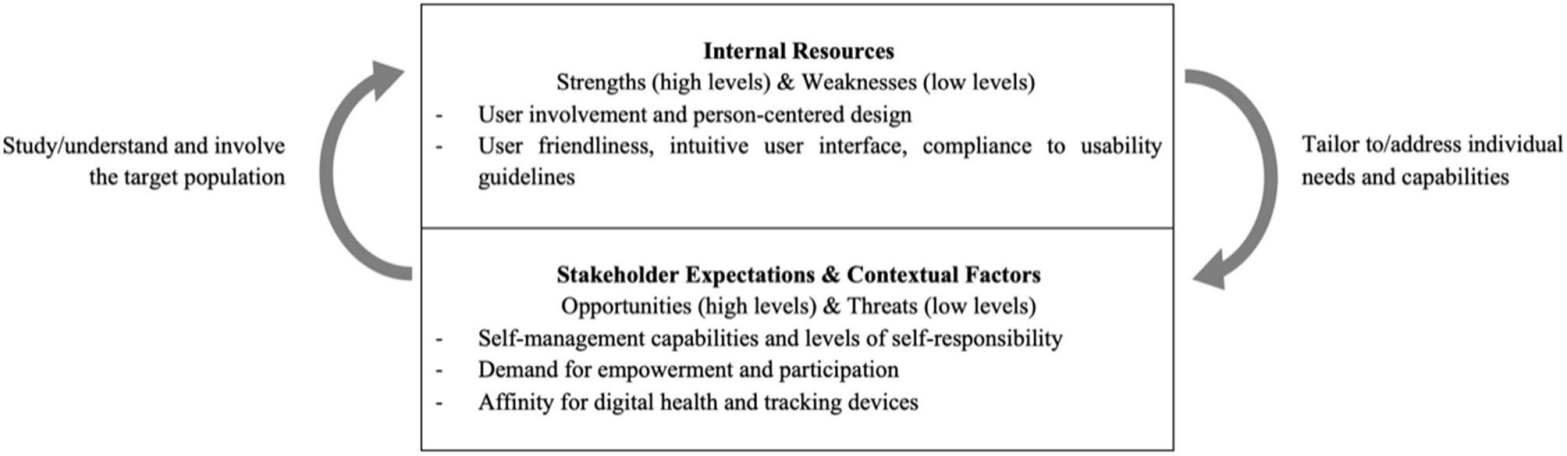
Dynamic SWOT Analysis for the Digital Management of Medication Adherence.

Key elements of this research and the interdependencies of the identified internal resources, stakeholder expectations, and contextual factors are illustrated in Figure 2. The willingness and ability of many patients to self-manage their therapies, their demand for more empowerment and participation, their affinity for digital technologies, and tracking individual health data seem to be promising contextual factors and stakeholder expectations for higher future engagement in digital health solutions. Nevertheless, the lower levels of engagement in digital solutions for patient self-management, empowerment, and education of more vulnerable population segments such as minorities, individuals with low educational levels, and older adults emphasize the hampering consequences of passive patient roles, insufficient health literacy, self-responsibility, and ability to self-manage medications. As an internal resource, the importance of focusing on user-friendliness, as well as compliance with existing guidelines for usability and accessibility of such digital health solutions, has been stressed. Therefore, interdependencies between the external and internal elements of the analysis emerge: as the ability to be self-responsible for medication management differs among patients, and insights into preferences regarding new technologies and their actual usage are still lacking, digital health solutions may lack a good fit for their target population if not successfully adapted to their specific needs. Because of the disadvantageous prerequisites of vulnerable groups, this may especially exclude population segments from the optimal usage that have a higher level of unmet medical needs. Nevertheless, if characteristics of the target population and especially needs of vulnerable individuals are carefully addressed, digital solutions can facilitate a participative way of service provision and motivate patients to take an active role in their treatments. Providing sufficient support and tailoring the intervention and information to patients' capabilities can help increase their self-responsibility.

Beyond the analysis of this research, the reorientation toward participation and involvement has been stated to be one of the most important shifts in health and medical care (56). Because of the advanced understanding of determinants of health, the boundaries of health systems become more fluid. It is more and more recognized that policy decisions as well as individual lifestyle choices have an impact on health (56). Within this emerging “health society,” “(…) the debate revolves around public and private responsibility, privatization and commercialization, health and wealth, empowerment and participation, and social inclusion and exclusion” (56). As health is presented as “doable,” citizens are demanded to participate in the health system and incorporate the dimension of health in their lifestyle decisions, while private and public services aim to provide support and navigation in decision-making processes (56). Various viewpoints that emerged within the interviews suggest that the boundaries of the health system and the obligation of its stakeholders to establish this self-responsibility throughout the society seem debatable. A proactive approach in health care provision was mentioned as an essential facilitator for more sustainable health systems and to accomplish unmet medical needs, especially against the background of challenges associated with the demographic change such as increasing chronic conditions and financial constraints. Efforts to enable citizens of all population segments to be more self-responsible and participative were mentioned to be essential. Especially interviewees with a strong quality and safety focus emphasized that it is a duty of health system actors to equip all citizens with the needed tools to make healthy lifestyle choices, to self-manage health conditions, and to provide a safety net if patients are unable to do so. On the other hand, it was questioned if societies and health systems should be held responsible for individuals' unhealthy behaviors and lifestyle choices. Especially interviewees also concerned with economic consequences of such policy decisions argued that inequalities are acceptable to a certain degree if the majority can realize benefits. Developments on these perspectives will be introduced and discussed in their application to the digital medication management innovation for the personalization of medications and the person-centered adherence management and similar digital solutions in the following.

Past development toward a more holistic definition of health and especially recent initiatives to construct a conceptual framework of health around “the ability to adapt and to self-manage” (57) reflect the attempt to incorporate the advanced understanding of biological, behavioral, and environmental determinants into health systems and services provided, exceeding the current World Health Organization definition (58). The new suggested conceptualization around adaptability and self-management may shift the focus of health policies on implementing more proactive and empowering measures (59) and promoting patient participation (60). Nevertheless, “the greater emphasis on individual responsibility in health care policy and public opinion might be considered to be in tension with the principles of solidarity and equal access to care (…)” (61). There is a tendency toward conceiving solidarity as a reciprocal concept that couples access to health services to a healthy lifestyle (61, 62). Policies shifting the responsibility toward citizens and patients may thus risk inequalities as a consequence of different levels of “the ability to adapt and self-manage” (59, 61).

The digital medication management innovation for the personalization of medications and the patient-centered adherence management should be placed in these contextual developments and expectations. One major aspect that emerges within the dimension of internal resources of the revised SWOT analysis is its potential to increase the adaptability and self-management capabilities of patients. The “precision dosing” feature provides personalized medication information and visualizes the position in the therapeutic window to empower patients to engage in their medication management actively. Further, the optimal use of medicines is accomplished by making adherence an integral part of personalized medicine. The identification of individual adherence behaviors as the basis for patient-centered adherence management, along with individual motivational factors and lifestyle factors, hence address potential determinants of inequalities. Nevertheless, sophisticated concepts are needed to increase the personalization and patient-centeredness of services. Results of the interviews as well as the supplementary literature review indicate that the vast potential of digital health solutions can only be unleashed if all aspects of the intervention, including the services provided, as well as the technologies used, are successfully tailored to the complex needs of the target population and allow for personalization on the individual patient level.

The presented SWOT analysis and the identified dynamic of facilitators and barriers indicate that digital solutions that empower and educate patients and increase their level of self-responsibility are promising approaches to pursue goals of regulators and policymakers. The risk of evolving inequalities has nevertheless to be carefully addressed. Efforts should especially include promoting the engagement of population segments that are the hardest to reach but are likely to benefit the most. Overall, it has been recognized that to unfold the potential that lies in digital solutions for the advancement of personalized medicine and adherence management, broad investments and full-scale implementation of such novel technologies are needed (20, 21).

Limitations of this research must be acknowledged. The literature search was not exhaustive and only performed on the PubMed database. Furthermore, the number of interviews was small, and the sample may not reflect the perspective of regulators and policymakers throughout Europe. Participants, however, covered various roles and responsibilities in organizations within the regulatory and policymaking field and were also concerned with policy decisions, implementation, and reimbursement in research. Nevertheless, the sample was limited to experts from only four western European countries and recruited with the convenient sampling approach. Further research should thus explore the perspectives of a broader and European-wide sample. Moreover, the involvement of all stakeholders emerged as an important aspect of successful design and implementation. This research was limited to the perspective of policymakers and regulators. It is thus suggested to investigate facilitators for and barriers to digital solutions for the personalization of medications and patient-centered adherence management from the perspective of other relevant stakeholders especially patients and health care professionals. It is hoped that this study provides a starting point for such research. In conclusion, this study suggests that, from the policymaker and regulator perspective, digital health solutions can be a facilitator to optimize the use of medicines and thus their efficiency while also promoting the implementation of new service structures and innovation especially around more participation. Different barriers were identified, with the most relevant from the perspective of policymakers and regulators, relating to the effects on equal accessibility and usage among all population segments. It is thus crucial to establish well-conceived development and implementation processes to also realize improvements in equality and solidarity within health systems.

## Data Availability Statement

The datasets presented in this article are not readily available because Interviewees would be identifiable in the interview transcripts. The detailed SWOT-analysis in the Supplementary Materials provides the anonymized and summarized data. Requests to access the datasets should be directed to Anna-Elisa Hein, anna.hein@student.maastrichtuniversity.nl.

## Ethics Statement

The studies involving human participants were reviewed and approved by Maastricht University ethical board at the Faculty of Health, Medicine and Life Science (REC Number: FHML/HPIM/2020.079). The patients/participants provided their written informed consent to participate in this study.

## Author Contributions

A-EH, BV, and MH designed the study reviewed the manuscript. A-EH collected, analyzed, and summarized the data and drafted the manuscript. BV reviewed the data collection and analysis. All authors were responsible for final approval of the version to be published.

## Conflict of Interest

BV is director general and a shareholder of AARDEX Group, Ltd., which is involved in developing, manufacturing, and marketing electronic medication-event monitors that measure, analyze, and facilitate adherence of patients and trial participants. The remaining authors declare that the research was conducted in the absence of any commercial or financial relationships that could be construed as a potential conflict of interest.
