# Supplementary material for: A Digital Innovation for the Personalized Management of Adherence: Analysis of Strengths, Weaknesses, Opportunities, and Threats
Source: Front Med Technol. 2020 Dec 14;2:604183. doi: 10.3389/fmedt.2020.604183 (PMC8757755; doi:10.3389/fmedt.2020.604183)
Supplement: Supplementary file 1 [file Data_Sheet_1.docx]

# Supplementary Material

Supplementary Material A: Search Strategies

| Search | Search Strategy | Date | Results | Included |
| --- | --- | --- | --- | --- |
| Regulators and policymakers | medical device regulation OR MDR OR device regulation OR Medical device OR Medical devices  AND  Europe OR European Union  AND  Authorization OR authorization OR registration OR market access OR HTA OR Health Technology assessment OR reimbursement  NOT  tuberculosis OR TB  AND  last 5 years | 10.04.2020 | 38 | 7  (24, 31-36). |
| Acceptance and uptake of digital self-management, empowerment, and education interventions | self-management OR self management OR empowerment OR education  AND  Acceptance OR usage OR uptake OR utilization  AND  equal access OR accessibility OR equality OR vulnerable  AND  eHealth OR mHealth OR mobile Health OR app OR apps  AND  last 5 years | 09.07.2020 | 35 | 16  (40-55) |

Supplementary Material B: Interview Structure

| Part 1 | |
| --- | --- |
| Aim | Verify fields of interest that emerged as priorities of interest of policymakers and regulators during the initial literature research and fill information gaps. |
| Key Points | - Roles, responsibilities, (eHealth/digitalization) projects - Practices, methods applied in regulatory/policymaking processes |
| *Example Questions* | - *Main Question: What are your roles and responsibilities in the regulatory/policymaking field?* - *Follow up question: What are currently major shortcomings or challenges in your field? What are your priorities?* - *Probes: Can you make an example how you address these challenges in your work?* |
| Part 2 | |
| Aim | Investigate strengths, weaknesses, opportunities, and threats for approaches to personalize medication and manage adherence, understand interactions of concepts, get insights in potential implications for successful implementation processes in the future |
| Key Points | - Strengths and weaknesses of personalizing medication and managing adherence by means of an app/medical device (positive and negative consequences of utilization of such approaches for stakeholders from the policymaker/regulator perspective) - Opportunities and threats for personalizing medication and managing adherence by means of an app/medical device (facilitating aspects and barriers within health care systems) - Readiness of health care systems |
| *Example Questions* | *Strengths and Weaknesses:*   - *Main question: Does the software/approach in your eyes have the potential to add value compared to the current practice of personalizing medication and managing adherence/could it lead to undesired consequences? How?* - *Follow up question: What outcomes of the features do you expect and what do you perceive as value adding/ disadvantageous?* - *Probes: Do you have examples of advantaged or disadvantages of current or similar approaches in practice?*   *Opportunities and Threats:*   - *Main Question: Do you think that HCS are ready to implement such digital innovations? How do you perceive the readiness of HCS?* - *Follow-up question: How do you perceive the readiness/willingness of different stakeholder groups (professionals, patients, payers, etc.) to integrate such digital/innovative approaches? What are other major barriers or facilitators for change/digital transformation/personalization* - *Have you experienced integration attempts or processes and can make an example of barriers and facilitators?* |

Supplementary Material C: Detailed SWOT-Analysis

| Strengths | |
| --- | --- |
| **Quality, safety, and effective patient access to personalized medicine** | |
| Facilitates better management of illnesses and medication and can reduce treatment complexity through personalization and information | - Can especially improve management of chronic illnesses - Provides information to better manage medication and avoid dose escalations - Can reduce treatment complexity |
| Improves quality, safety, effectiveness, and benefit-risk ratio of medications through personalization and feedback | - Increases safety, track quality defects - Improving efficiency (lack of efficiency considered as AE) - Optimizes benefit-risk ratio and clinical effectiveness, better use of therapies - Reduces/avoids hospitalization and other disease related consequences (increases QoL) |
| **New services structures, person-centeredness, and allocation of resources** | |
| Promotes patient-centered and multidisciplinary service provision and a new way of working between health professionals | - Can facilitate a new way of working between health professionals (e.g. lead to a more active role of the pharmacists in counselling patients and coordinate collaborative efforts of physicians and pharmacists, take workload from GPs) - Interesting for provider to know their patients' intake behavior/see their patients' adherence data |
| Facilitates a participative way of service provision and motivates patients to take an active role | - Provider can change the way they support their patients based on insights about causes auf non-adherence |
| Increases self-responsibility and capabilities of patients to understand and self-manage their medication through empowerment, education, and support | - Interesting for patients to know their intake behavior/see their adherence data - Inform patients about intake behavior to help them discover the causes for their non-adherence - Increased information enables patient to discuss medication and adherence with the physician - (Behavioral) support in building habits and routines increases adherence - Precision Dosing as a concept and adherence management through the chatbot (including empowerment and habit building) is innovative and promising - Although those with the largest scope for improvements may not be reached, still, many patients can benefit - Enables patients to be more self-responsible - Increases level of self-management and ability to make decisions regarding the intake behavior - Repetition, advices, feedback, motivation, insights into intake behavior increases understanding an ability to self-manage/adapt medication - Can support family members or care givers of patients with difficult medication management |
| Improves cost-effectiveness by reducing economic burden related to ineffective management of medication | - Reducing medicine expenditure, care costs, avoid economic burden (through adherence to expensive medication, less hospitalization, etc.) - Improve cost-effectiveness of the use of medicines - Good patient-centered approaches are likely to be cost-effective (business case usually follows automatically) |
| **Economic Growth and Competitiveness, Innovation, and Digital Transformation** | |
| Generates valuable data for improvements and further innovations | - Application can generate valuable data, retrieve RWE on the use of medicines - Generated data can help to further understand customer/patient needs - Can provide adherence information on the individual level |

| Weaknesses | |
| --- | --- |
| **Quality, safety, and effective patient access to personalized medicine** | |
| May add complexity to treatments and the management of medication | - May add complexity to the treatment, many drugs are not toxic or ineffective with small discrepancies in the time of consuming - Has to be ensured that results of the tool are compatible with other treatments - Can complicate the medication intake - Risk of negative impact on the patient's health (if self-management goes wrong) |
| May only be perceived necessary for a limited number of medications | - Has to proof preventive effect, that the benefit-risk ratio is really improved - Limited number of medications qualifying for Precision Dosing/TDM |
| Potential exclusion of (vulnerable) patient populations from the optimal use of medicines | - Potential exclusion of patient populations from optimal use of medicines (disabled, those who don't want it etc.) - May exclude those without affinity towards digital devices |
| **New services structures, person-centeredness, and allocation of resources** | |
| Ability to be self-responsible for medication management differs among patients | - Highly depends on the individual patient (need to analyze large amounts of data before), not every patient feels comfortable, could be nervous about self-managing their medication (e.g. elderly) - Who is suitable to receive this kind of information?) - Target population, patients with high non-adherence, those who would benefit the most may not be reached |
| Unclear preferences regarding the value of new technologies and hard to ensure their appropriate use | - Unclear how patients perceive the value of new technologies - Users my perceive user-friendliness differently - Hard to ensure adherence to the tool |
| May disturb the relationship between health care professionals and patients | - May disturb the relationship (patients may "lie" to the tool to be considered as "good patients") - May be perceived as a kind of monitoring policy |
| Implementation costs may exceed cost savings and integration requires additional training and collaborative efforts | - Cost of the tool itself, has to be affordable - Only if medication needs to be monitored (specific patient population), need for business case for which drugs the benefits outweighs the costs, not universally applicable/necessary - Integration requires special reimbursement scheme - Need for/dependent on collaborative efforts of pharmacists, physicians, nurses - Complexity requiring extra training for professionals - Integration requires additional education and training for providers |
| **Economic Growth and Competitiveness, Innovation, and Digital Transformation** | |
| Generated data may have irregularities that are hard to explain | - Hard to ensure that the patterns you see are true, "people (patients) do the strangest things"/approach irregularities with logical co-explanations (adds complexity) |

| Opportunities | |
| --- | --- |
| **Quality, safety, and effective patient access to personalized medicine** | |
| Increasing openness towards approaches to personalize treatments and development of supportive digital applications | - Complex medicines requiring tools like Precision Dosing (manufacturer of complex/high risk drugs will have to come up with concepts ensuring the safe and effective use of their medicines) - Need for devices to help patients managing their chronic conditions - There are people who think you should go towards precision medicine and use modern tools to communicate/early adopters (use models like segmented populations)/ (Young provider generation is more open for digital innovations, can advocate it) - Other approaches to reduce treatment complexity and SEs (improved drugs/monthly administration) may be preferred, the better the medication the less demand for adherence management and lifestyle changes |
| Increasing demand to improve adherence to medications with a narrow therapeutic window | - Increasing awareness of policymakers and initiatives to approach adherence problems (esp. In HCS or disease areas where the problem is huge/visible) - Much effort to assess and increase adherence for drugs where you need to monitor and ensure that everything is within a certain level |
| **New services structures, person-centeredness, and allocation of resources** | |
| New forms of collaboration and multidisciplinary approaches are emerging | - New forms of coordination and collaboration among providers are emerging, more multidisciplinary approaches, shifts in responsibilities (e.g. Providers make good experiences during Corona) - Discussions of adherence within the pharmacy space |
| Service structures to ensure long-term sustainability and patient-centeredness are gaining importance | - Direction towards more preventive approaches, policymakers see need for proactive measures to reduce waste - More patient-centered aspects are promoted - Policymakers try to give patients access to proactive services, collaborate with HTA, payers, and social security to ensure sustainability - Shifts in the way care is provided, more approaches and incentives for patient-centeredness (e.g. for diabetic patients) to use resources more effective (also through nudging), adherence is recognized as a huge problem in the management of chronic conditions - Direction towards building economically sustainable systems around patient needs, incentivizing multidisciplinary approaches (e.g. reimbursing pharmacists for advising patients) - Regulators can provide a kind of manual to HC professional as well as training material to minimize risks - Good patient-centered approaches are likely to be cost-effective (business case usually follows automatically) |
| Focus on methods to allocate financial resources in a cost-effective manner | - Comparative effectiveness approach, has to add something to be reimbursed to higher prices than the current standard (HTA level) - Costs have to be smaller than benefits - Alignment of requirements of HTA and regulators (objectively assess effect of drugs) to make health systems more sustainable |
| Increasing openness for digital innovations in health care | - Policymaker/researcher/Developer believe that new technologies can add value - Accelerated uptake of digital health tools due to Corona, increasing acceptance among providers and patients |
| Many patients are willing and able to self-manage their therapies and demand empowerment and participation | - Many patients already adjust their medication to their lives - Many patients are able to fully execute their own therapies - Patient empowerment is on policymakers’ agenda, need to involve patients more, directions towards more self-responsibility - Most patients are not intentionally forgetting their medication (might be open for support) - Other approaches (e.g. monthly injections) are more coercive, some patients may not feel comfortable with it |
| Affinity of many patients for digital tools, wearables, tracking of individual data | - Many patients are more advanced than professionals, need to progress at the same pace - Many patients are already using apps and wearables to support their health/positive attitude towards digital health, large group of patients with smartphones who can and may want to use such an app |
| **Economic Growth and Competitiveness, Innovation, and Digital Transformation** | |
| Ongoing improvements of the digital infrastructure and implementation processes for digital health care solutions | - Initiatives to improve EHR - Digital means are already being tested |
| Initiatives to generate health data and make best use of available sources | - Initiatives to make better use of available data like patient registries, generate more data in trials in clinical practice, RWE (to find out what happens at the home of the patient) - People may get more open to sharing information that are relevant to address public health questions due to corona crises (e.g. discussion around tracking app) - HTA agencies are forcing patients to provide information if they are paying for new expensive drugs - Better data and stronger evidence can increase awareness of the consequences on non-adherence - Regulators expect industry to support registries - Interest in adherence data within the individual phase |
| Opportunity of certification and development of registries to ensure high quality applications, to increase visibility, and guide uptake | - Opportunity to collaborate with regulators, openness/willingness to reimburse apps (e.g. DiGA in Germany) due to pressures towards digitalization - CE mark (and national registers of high-quality applications like DiGA) as kind of guarantee that reassures doctors that the data is validated and the app complies with all regulations, increases visibility among professionals - List of approved (and reimbursed) apps will be short |

| Threats | |
| --- | --- |
| **Quality, safety, and effective patient access to personalized medicine** | |
| High complexity of medications and uniqueness of patients | - Challenging to achieve appropriate concentration of active ingredient - Highly depends on the individual patient and the pathology - Payers/Policymakers/Regulators may expect more and more from pharmaceutical companies, manufacturer of complex/high risk drugs will have to come up with concepts ensuring the safe and effective use of their medicines |
| Suboptimal adherence behavior in treatments with a narrow therapeutic window leading to considerable disease-related consequences and adverse effects | - Patients where you expect adherence problems that are taking drugs with narrow therapeutic windows - Disease related consequences and AEs due to non-adherence |
| **New services structures, person-centeredness, and allocation of resources** | |
| Task-oriented service and remuneration structure and no clear strategy to improve and implement multidisciplinary and patient-centered structures | - No clear objectives to shift roles and responsibilities, e.g. pharmacists are not really considered as care givers, current repartition is not well organized (Task-centered service provision leads to low demand among providers), high unused resources (e.g. pharmacists) - Hard to enforce such a holistic management of diseases/medication - Regulators have no role in improving patient management, cannot enforce that patients are being monitored (only recommend based on patient reports, on effects of the drug that they see in patients) - Inertia to tackle adherence problems and implement proactive approaches esp. Due to the current organization of care/DoL between the providers - "Silostrukturen"/task-oriented /classical structure of HCS and therefore also reimbursement and incentive schemes (FFS) - Currently almost no incentives for providers to provide multidisciplinary care (e.g. pharmacists generate profit mainly through margin of products) |
| Passive role of the patient with insufficient health literacy, self-responsibility, and ability to self-manage medications | - Patients have low level of information; patient empowerment has to go hand in hand with the professional (esp. for those with low education level) - Patients have low level of self-management - Patients are not aware of their intake behavior/adherence/truly believe that they are adherent while they are not - little demand - Dishonesty due to patients wanting to be "good patients", highly dependent on the individual patient - Current approaches (patient information leaflets) are not sufficient, not understandable, need for innovative approaches (e.g. QR code with short video) |
| Determinants of adherence are complex and awareness among health care professionals of non-adherence is low | - Complexity of adherence and its determinants (depends on individual patients, mental issues, economic issues, etc.) - Provider (physicians) are not aware of their patients’ intake behavior/adherence (can lead to dose escalation)/hard to interfere in physicians' question routines - little demand - Often questioned if initiatives are really needed (esp. In HCS or disease areas where the problem is not huge/visible) |
| Conservative attitude towards digitalization among many health professionals and slow implementation processes | - Conservative attitude towards digital care among many providers/laggards, Provider are not trained for the use of digital tools (esp. Older generation), inertia to change, coexistence of different mindsets may make implementation challenging (Telemedicine due to corona has been challenging for some providers) - Need to stand out from other applications, it is impossible for GPs to follow the whole market - There are many adherence applications are on the market (competition and "noise" that leads to mistrust among professionals) - Implementation of digital innovations is very slow - Many patients are more advanced than professionals, need to progress at the same pace |
| Scarcity of financial resources, increasing complexity and costs of care, and low willingness to pay for new care models | - Financial crises with substantial financing problems for payers due to Corona, willingness to reimburse technological solution will decrease on the short and middle term - Discussion around what a health insurance has to pay for/current shift towards more self-responsibility and less solidarity - Increasing prices for new drugs (enormous economic burden for health systems) - Need for devices to help patients managing their medication or chronic conditions to decrease costs (also for societies as a whole due to e.g. absenteeism) - Less money is spent on prevention than on a curative approach, regulators cannot really enforce it, no comprehensive public health strategies in place - Payers willingness to pay for services (like adherence management) that have safe costs and only potential savings is low (also because there is a lot of "noise" around preventive/proactive approaches, not as visible as need for curative services) |
| **Economic Growth and Competitiveness, Innovation, and Digital Transformation** | |
| Slow progress in the infrastructure for digital solutions and data governance regulations | - Still no widely shared EHR - Data protection is a problem in many competing applications - Many applications have data protection problems, market sorts itself - High and complex data protection standards/requirements |
| Highly regulated and bureaucratic market access | - Separate market access process makes it less attractive for manufacturers to couple drug and device - Bureaucratic/highly regulated market access procedure (some innovations are already outdated when approved/implemented) |
| Insufficient generation and usage of health (and adherence) data | - Insufficient data generation tools, much unrevealing data from observing (e.g. pharmacy data), big challenge to have realistic figures - Current research practice requires that patients give informed consent so their data can be used, only able to study the "good" patients because they usually give consent, hard to get information for "bad" patients, non-adherent patients usually don’t want to be monitored (e.g. patients with mental diseases may be afraid they are monitored by the government) - Adherence uninformed trials accepted as current practice |
